# Inflammatory arthritis—the end of the golden age

**DOI:** 10.1093/rap/rkac015

**Published:** 2022-02-24

**Authors:** James A Stanway, David Walker

**Affiliations:** 1 Translational and Clinical Research Institute, Newcastle University, Newcastle; 2 Rheumatology Department, Northumbria Healthcare Trust, Northumbria, UK

Dear Editor, Over the years there has been a history of drugs used for rheumatic conditions, which patients have found useful and which could be continued on an individual risk–benefit basis, being withdrawn from use. Sometimes this has been attributable to toxicity concerns for the user (e.g. lumiracoxib). Sometimes it has been for wider public health issues, such as toxicity in overdose (e.g. co-proxamol) [1]. For others, it has been a simple commercial decision by the manufacturer because it is no longer profitable to market (e.g. auranofin and benoral). It has been suggested that this should not be allowed under the licence and that supply should be maintained for patients who wish to continue and where their physician believes that the risk–benefit is in favour of continuing [2]. In 2019, i.m. gold was withdrawn worldwide. This, we were told, was because the raw materials were no longer available [3]. However, it seems inconceivable that there was not an economic factor in this decision. If we could make sodium aurothiomalate in the 1930s, then presumably we could still make it if there were to be sufficient incentive.

Before the widespread use of MTX, i.m. gold was commonly used for the management of inflammatory arthritis [4]. Patients who were users of i.m. gold in 2019 had either been on it for many years (and for whom it was a very satisfactory drug) or they had been started on it more recently, chosen for its toxicity profile in patients who had failed many other drugs or because it was not subject to restrictive National Institute for Health and Care Excellence guidance

To evaluate the effect of its withdrawal, we searched our departmental database in Northumbria Healthcare Trust for users of i.m. gold in July 2019 and recorded changes to their treatment regimen and disease control up to July 2021. From a cohort of ∼4750 (RA: 2500; PsA: 1700; axial spondylitis: 550), we identified 37 patients receiving gold: 32 for RA; 4 for PsA and 1 for axial spondylitis. Of these, 28 were taking gold as monotherapy (3 of whom also required regular CS) and the rest in combination with another DMARD (4 MTX, 3 SSZ, 1 LEF and 1 rituximab). Records of the indication for gold over alternatives were not available in all cases, but concern regarding frequent chest infections was a common theme and clearly documented as a contraindication to biologics in five cases. We attempted follow-up during autumn 2021, ∼2 years after withdrawal, to observe patient progress and changes in medication.

Follow-up has been impacted by the coronavirus disease 2019 pandemic, with 11 patients yet to be reviewed since cessation of therapy. It is expected that this reflects the absence of flare in these cases, because the Northumbria Rheumatology service operates an effective emergency helpline. Amongst those taking gold as part of combination therapy, no new DMARDs have been initiated, and only one patient appeared to have a deterioration in disease control. Amongst the 28 patients receiving gold monotherapy, 13 have commenced new treatments, with 5 commencing biologic/targeted synthetic DMARDs. Of the remaining 15 patients, 3 have experienced a deterioration in their disease control requiring additional CSs but are yet to be established successfully on a replacement DMARD; 6 have stable disease, and 6 are yet to be seen.

In total, 35% of patients commenced a new DMARD, and an additional 11% experienced a flare requiring CS. Reassuringly, however, over half the patients demonstrated stable disease in the absence of further therapy. This suggests that a proportion of patients on long-term treatments do not need them and highlights the need to consider treatment tapering or withdrawal in those with stable disease. This should be a shared decision between patient and physician, rather than a result of withdrawal by the manufacturer. However, almost half of our patients who were stable on i.m. gold have suffered increased disease activity or required a change of therapy as a result of this withdrawal, and some are still not on satisfactory replacement. With many superior agents available today, gold, appropriately, does not feature in modern treatment algorithms. However, it still proved to be very satisfactory drug for the small but significant proportion of patients taking it, some of whom struggle to find a suitable alternative. The regulators of the licensing of medications should consider the consequences of unilateral withdrawal and could perhaps prevent such occurrences in the future.

*Funding:* No specific funding was received from any bodies in the public, commercial or not-for-profit sectors to carry out the work described in this article.

*Disclosure statement:* The authors have declared no conflicts of interest.

## Data availability statement

Anonymized data are available from the authors on request.
